# Mice with Hypomorphic Expression of the Sodium-Phosphate Cotransporter PiT1/Slc20a1 Have an Unexpected Normal Bone Mineralization

**DOI:** 10.1371/journal.pone.0065979

**Published:** 2013-06-13

**Authors:** Annabelle Bourgine, Paul Pilet, Sara Diouani, Sophie Sourice, Julie Lesoeur, Sarah Beck-Cormier, Solmaz Khoshniat, Pierre Weiss, Gérard Friedlander, Jérôme Guicheux, Laurent Beck

**Affiliations:** 1 Institut National de la Sante et de la recherche Medicale, U791, LIOAD, STEP group "Skeletal Tissue Engineering and Physiopathology”, Nantes, France; 2 Université de Nantes, UFR Odontologie, Nantes, France; 3 Institut National de la Sante et de la recherche Medicale, U845, Centre de Recherche Croissance et Signalisation, Paris, France; 4 Université Paris Descartes, Paris, France; Institut de Génomique Fonctionnelle de Lyon, France

## Abstract

The formation of hydroxyapatite crystals and their insertion into collagen fibrils of the matrix are essential steps for bone mineralization. As phosphate is a main structural component of apatite crystals, its uptake by skeletal cells is critical and must be controlled by specialized membrane proteins. In mammals, *in vitro* studies have suggested that the high-affinity sodium-phosphate cotransporter PiT1 could play this role. *In vivo*, *PiT1* expression was detected in hypertrophic chondrocytes of murine metatarsals, but its implication in bone physiology is not yet deciphered. As the complete deletion of *PiT1* results in embryonic lethality at E12.5, we took advantage of a mouse model bearing two copies of *PiT1* hypomorphic alleles to study the effect of a low expression of *PiT1* on bone mineralization *in vivo*. In this report, we show that a 85% down-regulation of *PiT1* in long bones resulted in a slight (6%) but significant reduction of femur length in young mice (15- and 30-day-old). However, despite a defect in alcian blue / alizarin red S and Von Kossa staining of hypomorphic 1-day-old mice, using X-rays micro-computed tomography, energy dispersive X-ray spectroscopy and histological staining techniques we could not detect differences between hypomorphic and wild-type mice of 15- to 300-days old. Interestingly, the expression of *PiT2*, the paralog of *PiT1*, was increased 2-fold in bone of *PiT1* hypomorphic mice accounting for a normal phosphate uptake in mutant cells. Whether this may contribute to the absence of bone mineralization defects remains to be further deciphered.

## Introduction

Bone mineralization is mainly orchestrated by osteoblasts through a complex and spatially regulated process initiated by the synthesis of type I collagen rich extracellular matrix (MEC) that will ultimately be the place of carbonated hydroxyapatite deposition. Calcium (Ca) and phosphate (Pi) that are essential for the formation of hydrocarbonated apatite come from blood serum that represents the main source of ions in the vertebrate body [1–3]. However, the mechanisms and cellular processes through which Ca and Pi are translocated from the serum to the site of calcification within the MEC are still under investigation. To date, three different modes have been described to explain mineral deposition into collagen matrices: (i) crystals deposition can occur without intervention of intracellular processes from solution by charged non-collagenous proteins in the collagen spaces [4]; (ii) matrix vesicles (MV) may bud from the plasma membrane, accumulate ions extracellularly and serve as primary nucleation sites [5,6]; and (iii) crystallization of hydroxyapatite may arise from a transient amorphous mineral precursor deposited within the collagen gap zones [1,7,8]. All three processes participate to the establishment of the mineralization front, wherein crystals of hydroxyapatite represent the main mineral component. Due to its structural role in the apatite crystal, Pi is considered as a major factor regulating the mineralization process [9]. Its uptake by the osteoblasts lining the bone surface, or by the mineralizing growth plate chondrocytes is usually considered as a pre-requirement to the mineralization process that must be tightly controlled by specialized membrane proteins [10].

In mammals, members of the SLC34 family of sodium-coupled Pi transporters were mainly associated with renal- or intestinal-related functions, in accordance with their tissue distribution but were not yet proved to have a relevant role in bone physiology [11–13]. On the other hand, PiT1, a member of the SLC20 family of sodium-coupled Pi transporters has long been recognized as a putative important Pi transporter in bone. Significant enough, *PiT1* was shown to be upregulated in osteoblastic cells at the time of onset of mineralization [14]. The expression of PiT1 was also correlated to the differentiation of ATDC5 and CFK2 chondrogenic cell lines, suggesting that it may represent the main Pi transporter present in these cells. Phosphate transport studies conducted in ATDC5-derived MVs were indicative of the existence of a Na-dependent Pi transport system with close characteristics to the one of PiT1 [15,16]. Consistent with this observation, *PiT1* expression was detected in early hypertrophic chondrocytes of murine metatarsals during their differentiation, suggesting a potential role for PiT1 in mineralization [17]. This putative role is supported by early studies describing the regulated expression of PiT1 by factors having important roles in bone, such as insulin-like growth factor-1 [18], transforming growth factor-alpha [19], epinephrine [20], bone morphogenetic protein 2 [10] and Pi [21–23]. Yoshiko and colleagues suggested that PiT1-mediated Pi uptake was required for osteoid mineralization in mice, and hypothesized that a defect in endogenous Pi-sensing/uptake system involving PiT1 could be sufficient to cause mineralization disorders independently of systemic Pi concentrations [24].

Although these *in vitro* studies are in favor of a putative role of PiT1 during bone mineralization, the precise implication of PiT1 in bone physiology is still lacking. The generation of a mouse model deficient in PiT1 revealed that this protein was essential to normal liver development since *PiT1*-null mice died around mid-gestation from severe anemia arising from liver development defects [25]. As this model could not be used to investigate the role of PiT1 in bone physiology, we took advantage of a mouse model bearing two copies of *PiT1* hypomorphic alleles (*PiT1* ^*neo/neo*^) resulting in the expression of only 15% of wild-type *PiT1* mRNA [25] to study the physiological relevance of PiT1 in bone growth and mineralization.

## Materials and Methods

### Animals and Ethics Statement

Animal care and maintenance were provided through the University Paris, Descartes accredited Animal Facility at Necker Faculty of Medicine (Paris). All procedures were approved by the Animal Care and Use Committee of the University Paris, Descartes (Comité Régional d’Éthique pour l’Expérimentation Animale, Ile de France – Réné Descartes), registered number P2.LB.112.09. Mice were maintained on rodent laboratory chow (Special Diet Services, Witham, Essex, UK) containing 0.73% calcium, 0.52% phosphate and 600 IU/kg of vitamin D3. Hypomorphic *PiT1* ^*neo/neo*^ mice were generated previously in our lab [25]. These mice bear two copies of a *PiT1* ^*neo*^ allele containing a neomycin resistance (neo) cassette and *loxP* sites flanking *PiT1* exon 5. We showed that transcription from the *neo* allele resulted in aberrantly as well as correctly spliced transcripts [25]. Quantification of both transcripts in E11.5 total embryos demonstrated that the *PiT1* ^*neo*^ allele is a hypomorphic allele leading to the expression of only 15% of correctly spliced wild-type *PiT1* mRNA levels.

### Cartilage and Bone Staining

For staining and visualization of whole skeletons, mice were dissected and skeletons were stained with alizarin red S and alcian blue 8G (Sigma), as previously described [26].

### Bone Histomorphometry by X-Rays Micro-Computed Tomography (µCT)

Histomorphometry was conducted on femurs from *PiT1* ^*+/+*^ and *PiT1* ^*neo/neo*^ mice from 15 to 300 days of age. The femurs were placed on a cylindrical sample holder in air in a high-resolution X-rays µCT system (Skyscan 1072, Kartuizersweg, Belgium). Using 61 kV and 148 µA, the µCT registers a series of radiographic images of a bone sample in rotation around a 180° axis with an angle step of 0.68°. Three dimensional images of bones were acquired in all spatial directions. The NRECON software (Skyscan) allows obtaining sections perpendicular to the X-ray images obtained with the µCT. The trabecular bone was separated from the cortical bone with manual drawn contours on a region of interest of 2 mm extended from the growth plate. Calculation of trabecular bone microarchitecture parameters (Bone volume fraction: BV/TV, Trabecular number: Tb.N, Trabecular thickness: Tb.Th, Trabecular separation: Tb. Sp) were performed using CTAn software (Skyscan).

Experiments were performed separately on male and female mice. As we did not observe any significant difference between males and females, data presented in this report include both male and female mice regardless of the gender.

### Histological Analysis

Femurs from 1-day-old to 300-day-old *PiT1* ^*+/+*^ and *PiT1* ^*neo/neo*^ mice were fixed in 4% paraformaldehyde for 24 hours, dehydrated in graded ethanol and embedded in glycol methacrylate. Longitudinal sections of 5 µm-thick were performed with a hard tissue microtome (Leica polycut SM 2500, Wetzlar, Germany) every 100 µm (or 50 µm for 1-day-old mice), collected on polylysine-coated slides and stained with Von Kossa, Goldner’s trichrome or Movat’s pentachrome staining using the Shandon Varistain Gemini ES system (Thermo Scientific, Courtaboeuf, France). Images were taken using a Zeiss Axioplan 2 microscope and Zen Lite software.

### Energy Dispersive X-Ray Spectroscopy (EDX)

An EDX analysis was performed on 1- to 300-day-old *PiT1* ^*+/+*^ and *PiT1* ^*neo/neo*^ femurs to analyze the surface composition. After fixation, femurs were carbon-coated (JEOL JEE 4B, Tokyo, Japan) and analyzed using a scanning electron microscope (LEO 1450VP, Zeiss, Weimar, Germany) fitted with an EDX system (INCA software, Oxford, England) at 15 kV. The main components of bone tissue (calcium, phosphate, magnesium, sodium and oxygen) were measured and calcium/phosphate (Ca/P) ratio was determined. Three femurs per group were studied and approximately twenty EDX microanalyses were performed for each femur.

### Real-Time PCR

Total RNA was extracted from frozen mouse tibias using the NucleoSpin RNA II kit (Macherey-Nagel, Düren, Germany), and was reverse transcribed with Affinity Script reverse transcription (Agilent) as per manufacturers’ instructions. Real-time PCR was performed on a Mx3000P System (Stratagene) using Brilliant III Ultra Fast SYBR QPCR Master Mix (Agilent Technologies). The following temperature profile was used: denaturation at 95°C for 3 minutes, amplification during 40 cycles of 5 seconds at 95°C and 20 seconds at 60°C, followed by a step at 95°C for 1 minute and 65°C for 30 seconds. Expression of target genes were normalized to *Gapdh* expression levels and the ∆Ct (cycle threshold) method was used to calculate relative expression levels as previously described [27]. The sequences of primers used in this study are listed in Table 1.

**Table 1:** Murine-specific primers used for RT-PCR analyses.

Gene	Sense (5’–3’)	Antisense (5’–3’)
*Gapdh*	GAAGGTCGGTGTGAACGGAT	CGTTGAATTTGCCGTGAGTG
*PiT1*	TGTGGCAAATGGGCAGAAG	AGAAAGCAGCGGAGAGACGA
*PiT2*	CCATCGGCTTCTCACTCGT	AAACCAGGAGGCGACAATCT
*Npt1*	TCCTGGAAGAAGGAAGGGCCGT	CAGGGAAGGACCCCAAAAGCCC
*Npt2a*	AGCCCCAGGGAGAAGCTATC	CCACAGTAGGATGCCCGAGA
*Npt2b*	CAGGACACTGGGATCAAATGG	GAAGGCGCTGCTCAGTACATC
*Npt2c*	CAGCCCTGCAGACATGTTAAT	GCACCAGGTACCACAGCAG

### Serum Analysis

Concentrations of plasma phosphate and calcium were determined in 15- to 300-day-old *PiT1* ^*+/+*^ and *PiT1* ^*neo/neo*^ mice. Blood samples were collected at the retro bulbar eye level in dry tubes, placed on ice, centrifuged and serum was collected. The serum samples were analyzed in the Centre d’Explorations Fonctionnelles Intégrées of the Faculty of Medicine Xavier Bichat (Paris) using a Olympus AU400 clinical chemistry analyzer.

### Embryonic Fibroblasts Culture and Analysis

Isolation of mouse embryonic fibroblasts (MEFs) was performed as previously described [25] and cultured in DMEM supplemented with 10% FBS in 5% CO_2_ and a humidified atmosphere. For phosphate uptake measurements, 25,000 MEFs were seeded in triplicates in 24-well plates and uptake was performed three days later as previously described [28]. Apparent affinity constant (K_m_) and maximal transport rate (V_max_) were calculated by nonlinear curve fitting, assuming Michaelis-Menten kinetics.

### Statistical Analysis

Data are expressed as mean ± SEM. Statistically significant differences between *PiT1* ^*+/+*^ and *PiT1* ^*neo/neo*^ mice were evaluated using the Mann & Whitney’s method. A p value of less than 0.05 was considered significant.

## Results

### Growth of hypomorphic *PiT1* ^*neo/neo*^ mice

**Figure 1:**
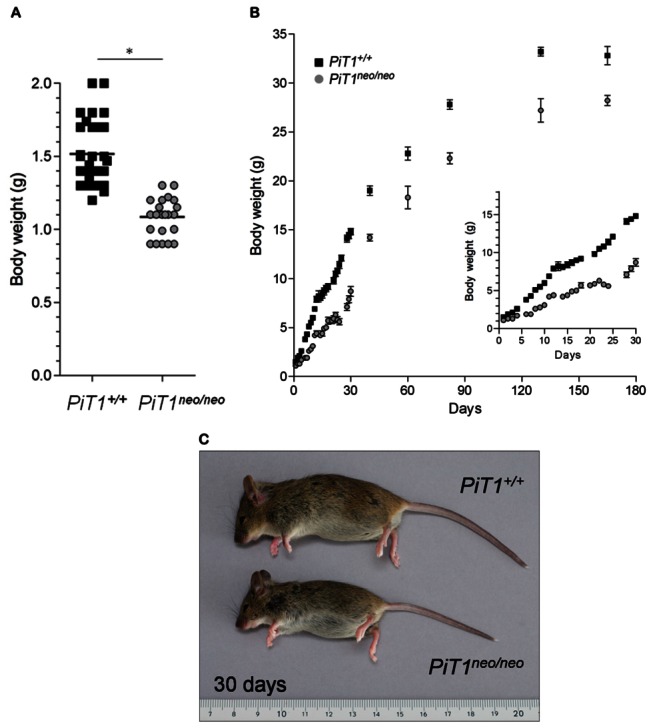
Growth of hypomorphic *PiT1* ^*neo/neo*^ mice. (**A**) Body weight (g) of 1-day-old *PiT1* ^*+/+*^ and *PiT1* ^*neo/neo*^ mice. (**B**) Evolution of body weight of *PiT1* ^*+/+*^ (n = 3 to 19) and *PiT1* ^*neo/neo*^ (n = 3 to 10) female mice from 1 to 165 days. Inset: growth curve of mice from 1 to 30 days of age. Data represent mean ± SEM. Statistical analysis was carried out by Mann & Whitney’s method. (**C**) Growth appearance of *PiT1* ^*+/+*^ and *PiT1* ^*neo/neo*^ mice at 30 days of age.

We previously reported that homozygous *PiT1* ^*neo/neo*^ mice were growth retarded at birth and up until the age of 2 weeks [25]. We confirmed this data on a much larger number of mice and monitored the weight of wild-type and *PiT1* ^*neo/neo*^ mice on a much larger time-frame, up until the age of 165 days. The mean weight of *PiT1* ^*neo/neo*^ 1-day-old mice (n = 24) presented a 29% inhibition compared to *PiT1* ^*+/+*^ 1-day-old mice (n = 33) (Figure 1A). The weight difference, corresponding to a 36% inhibition, was maximal at 30 days of age for both male and female mice (Figure 1B) and persisted up until 6 months of age (Figure 1B) indicating that hypomorphic *PiT1* ^*neo/neo*^ mice are smaller than *PiT1* ^*+/+*^ mice throughout their entire life.

### Skeletal mineralization in hypomorphic *PiT1* ^*neo/neo*^ mice

**Figure 2:**
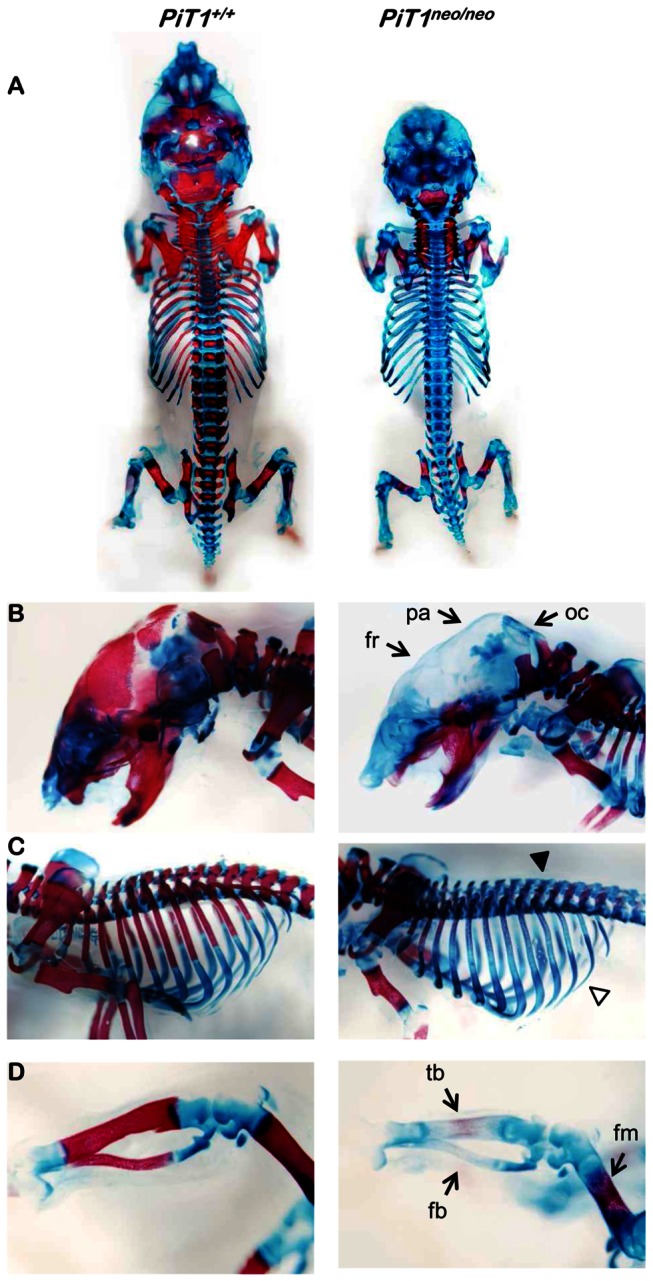
Alcian blue / alizarin red S double staining of the skeleton of 1-day-old *PiT1* ^*+/+*^ and *PiT1* ^*neo/neo*^ mice. (**A**) Whole skeleton staining of *PiT1* ^*+/+*^ and *PiT1* ^*neo/neo*^ mice. (**B**) Magnification on the skull: the frontal (fr), parietal (pa) and occipital (oc) bones are not mineralized in *PiT1* ^*neo/neo*^ 1-day-old mice. (**C**) *PiT1* ^*neo/neo*^ 1-day-old pups present a significant delay of mineralization at the ribs (white arrow head) and the spine (black arrow head). (**D**) Magnification on the hind limbs: the tibia (tb) and the fibula (fb) of *PiT1* ^*neo/neo*^ 1-day-old mice are not mineralized, and a retardation of mineralization is observed in the femur (fm).

We already documented that an hypomorphic expression of *PiT1* has no detectable consequences on early skeletal development [25]. However, we noticed that 1-day-old *PiT1* ^*neo/neo*^ mice may present with an impaired skeletal mineralization [25]. As confirmed and illustrated in Figure 2A, the whole skeleton of 1-day-old *PiT1* ^*neo/neo*^ mice was less mineralized as evidenced by the fainter alizarin red S staining, as compared to *PiT1* ^*+/+*^ littermates. Regional differences were seen in alizarin red S staining intensity, particularly on the skull, the frontal (fr), parietal (pa) and occipital (oc) bones which were not mineralized in *PiT1* ^*neo/neo*^ 1-day-old mice (Figure 2B). *PiT1* ^*neo/neo*^ 1-day-old pups also exhibited a significant delay of mineralization at the ribs and the spine as shown in Figure 2C. Furthermore, the tibia (tb) and the fibula (fb) of *PiT1* ^*neo/neo*^ 1-day-old mice were not mineralized, and a retardation of mineralization is observed in the femur (fm) (Figure 2D).

**Figure 3:**
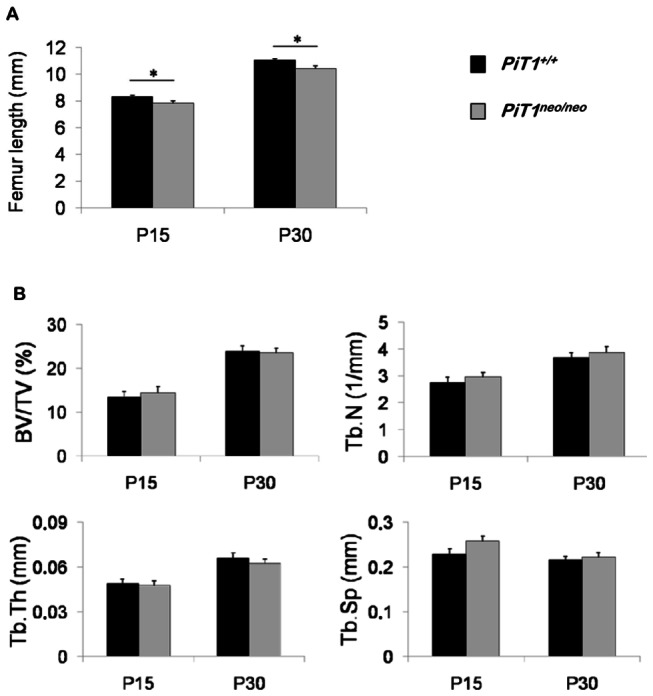
Histomorphometric analysis of femurs from *PiT1* ^*+/+*^ and hypomorphic *PiT1* ^*neo/neo*^ mice. (**A**) Femur length of 15- and 30-day-old *PiT1* ^*+/+*^ and *PiT1* ^*neo/neo*^ mice. At 15 days of age, there is a significant difference between the femur length of *PiT1* ^*+/+*^ (n = 11) and *PiT1* ^*neo/neo*^ (n = 10) mice. This difference persists at 30 days of age (*PiT1* ^*+/+*^: n = 18; *PiT1* ^*neo/neo*^: n = 19). (**B**) Essential histomorphometric parameters to describe trabecular bone morphometry in 15- and 30-day-old *PiT1* ^*+/+*^ and *PiT1* ^*neo/neo*^ femurs: the bone volume fraction (BV/TV), the trabecular number (Tb.N), the trabecular thickness (Tb.Th) and the trabecular separation (Tb. Sp). Data are represented as mean ± SEM. The analysis was repeated in 8 to 21 male and female mice in each group. Statistical analysis was carried out by Mann & Whitney’s method.

To better assess the effect of a reduced expression of *PiT1* on bone mineralization, we performed a histomorphometric study on isolated femurs. As shown in Figure 3A, femurs isolated from 15-day-old *PiT1* ^*neo/neo*^ mice (n = 10) were shorter from the femoral head to the distal condyle than femurs from *PiT1* ^*+/+*^ mice (n = 11). This 6% difference between wild-type and mutant femurs persisted at 30 days of age (*PiT1* ^*+/+*^ mice, n = 18; *PiT1* ^*neo/neo*^ mice, n = 19) indicating that longitudinal bone growth was impaired. The shorter size of femurs is consistent with the reduction in gross size of hypomorphic *PiT1* ^*neo/neo*^ mice compared with *PiT1* ^*+/+*^ mice (Figure 1). Bone morphology and microarchitecture were assessed by µCT. Results showed that there was no significant difference in the four essential variables describing trabecular bone morphometry [29] which are the bone volume fraction (BV/TV), the trabecular number (Tb.N), the trabecular thickness (Tb.Th) and the trabecular separation (Tb. Sp) between femurs from 15- and 30-day-old *PiT1* ^*+/+*^ and *PiT1* ^*neo/neo*^ mice (Figure 3B). As *PiT1* ^*neo/neo*^ mice present a smaller weight, they also have a smaller bone volume (BV). However, when the BV values were normalized with the total volume (TV), the BV/TV ratios were not statistically different between *PiT1* ^*+/+*^ and *PiT1* ^*neo/neo*^ mice.

**Figure 4:**
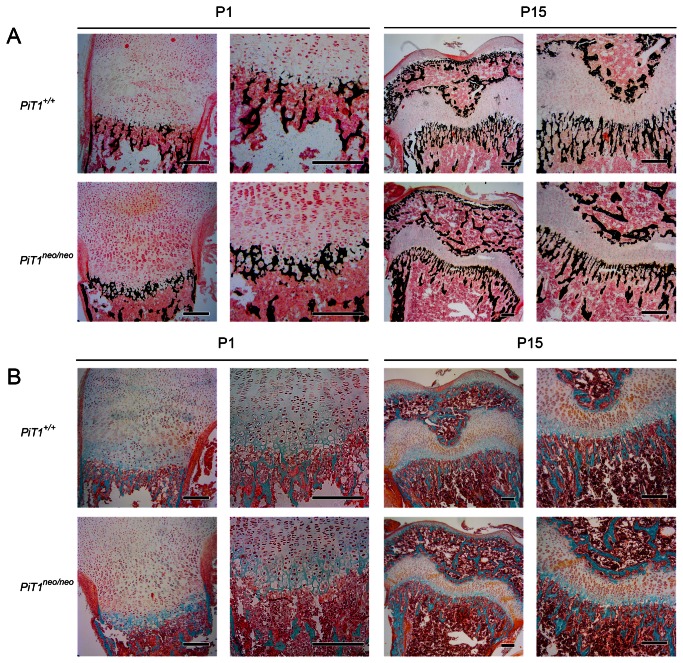
Von Kossa and Goldner’s trichrome histological staining of femur sections from *PiT1* ^*+/+*^ and hypomorphic *PiT1* ^*neo/neo*^ mice. Femurs from 1- (P1) and 15- (P15) day-old *PiT1* ^*+/+*^ and *PiT1* ^*neo/neo*^ mice were fixed in 4% paraformaldehyde and stained using Von Kossa (**A**) or Goldner’s trichrome (**B**) staining method. Using Von Kossa staining, the mineral deposition is stained in black. The Goldner’s trichrome reveals the glycoaminoglycan matrix in blue-green. With these two staining, the osteoid is stained in red. Bars represent 200 µm.

**Figure 5:**
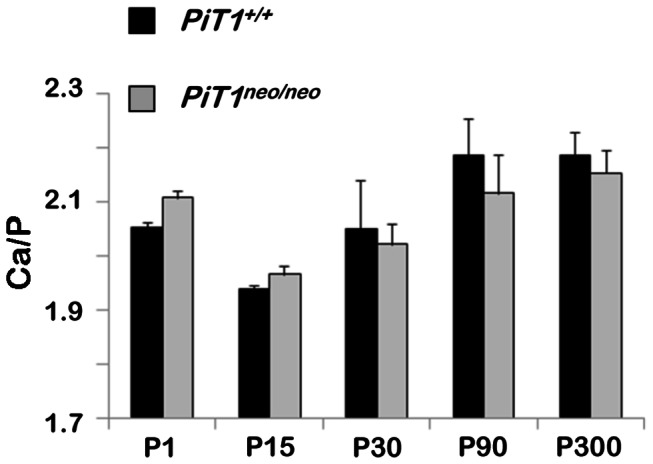
Energy dispersive X-ray spectroscopy (**EDX**) analysis of femurs from *PiT1* ^*+/+*^ and hypomorphic *PiT1* ^*neo/neo*^ mice. Ca/P ratio in 1- to 300-day-old *PiT1* ^*+/+*^ and *PiT1* ^*neo/neo*^ femurs. Data are represented as mean ± SEM. The analysis was repeated in 3 mice femurs in each group with twenty EDX microanalyses per femur. Statistical analysis was carried out by Mann & Whitney’s method.

When analyzing the histomorphological parameters of femurs from 1-day-old littermates using µCT, we had trouble separating the cortical bone from the trabecular region, which led us to perform histological stainings of bone sections, as well as an energy dispersive X-ray spectroscopy (EDX) analysis. Consistent with alcian blue / alizarin red S staining of whole skeletons, the Von Kossa black staining (Figure 4A) of femur sections from 1-day old *PiT1* ^*neo/neo*^ mice was fainter as compared to wild-type mice, suggesting a decrease in mineral deposition. Consistent with this result, when using the Goldner’s trichrome staining technique (Figure 4B), we could evidenced that the blue stain revealing the cartilage was slightly more increased in femur sections from 1-day old *PiT1* ^*neo/neo*^ mice as compared to wild-type mice. However, when analyzing bone sections from older animals (from 15-days old to 300-days old), no differences could be seen between *PiT1* ^*+/+*^ and *PiT1* ^*neo/neo*^ mice (Figure 4 and Figure S1). To determine whether the difference in mineral deposition seen in 1-day old femurs could be attributed to composition differences arising from a decrease in Pi availability, the surface composition of bone was measured using EDX analysis (Figure 5). Interestingly, expressing the results as Ca/P ratios, representing a sensitive measure of bone mineral changes, we could not evidence any significant difference between *PiT1* ^*+/+*^ and *PiT1* ^*neo/neo*^ mice from 1 to 300 days of age. Similarly, no difference was observed in magnesium, sodium and oxygen content (data not shown), meaning that the global composition of bone (and not only the one of mineral phase) was not different between wild-type and mutant mice.

### Expression of sodium-dependent phosphate transporters in tibias of hypomorphic *PiT1* ^*neo/neo*^ mice

**Figure 6:**
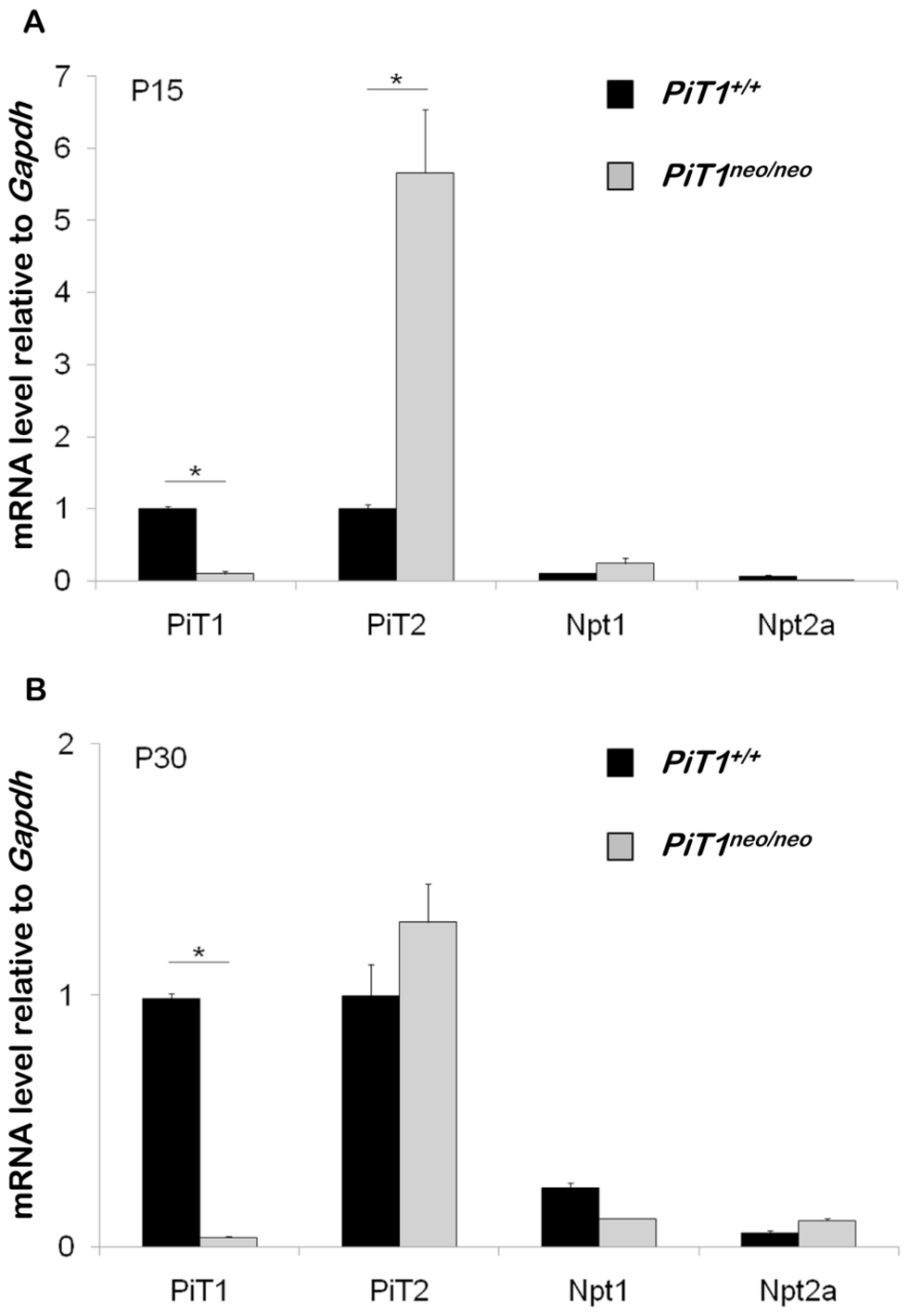
Expression of sodium-dependent phosphate transporters in tibias of hypomorphic *PiT1* ^*neo/neo*^ mice. Total RNA was extracted from tibias of 15- (**A**) and 30-day-old (**B**) *PiT1* ^*+/+*^ (black bars) and *PiT1* ^*neo/neo*^ (grey bars) mice and analyzed by RT-PCR for the expression of *PiT1*, *PiT2*, *Npt1*, *Npt2a*, *Npt2b* and *Npt2c*. Results are reported after normalization to the expression of *Gapdh*. Data are represented as mean ± SEM from analysis of 6 mice per group. Statistical analysis was carried out by Mann & Whitney’s method.

Expression analysis of *PiT1* in the *PiT1* ^*neo/neo*^ E11.5 embryos was already shown to result in the expression of only 15% of wild-type *PiT1* mRNA due to an abnormal splicing of the *PiT1* ^*neo*^ allele [25]. On the other hand, the level of *PiT1* expression from the *PiT1* ^*neo*^ allele of young or adult mouse bone tissue is not known. To quantify the *PiT1* expression specifically in bone, we performed RT-PCR analysis on RNAs extracted from tibias of 15- and 30-day-old *PiT1* ^*+/+*^ and *PiT1* ^*neo/neo*^ mice. As shown in Figure 6, the expression of *PiT1* in tibias of *PiT1* ^*neo/neo*^ mice was significantly reduced to about 15% of wild-type *PiT1* expression. Similar results were obtained when measuring the *PiT1* expression from liver and kidneys of normal and mutant mice (data not shown), indicating that the expression of *PiT1* from the *PiT1* ^*neo*^ allele did not differ between tissues and occurred also in bone.

To determine whether a down regulation of *PiT1* expression would lead to a change in the expression of other known phosphate transporters, we evaluated the expression of *PiT2, Npt1, Npt2a, Npt2b* and *Npt2c* in bone. At 15 days of age, the expression of *PiT2* was significantly increased in tibias of hypomorphic *PiT1* ^*neo/neo*^ mice (Figure 6A). This result is consistent with the observed *PiT2* overexpression in *PiT1* ^*neo/neo*^ E11.5 whole embryos in which *PiT1* was inhibited at 85% [25]. Interestingly, the *PiT2* overexpression in bone was no longer observed at 30 days of age in bone (Figure 6B). The expression of *Npt2b* and *Npt2c* was not detected (data not shown) whereas *Npt1* and *Npt2a* were weakly expressed in the bone of *PiT1* ^*+/+*^ and *PiT1* ^*neo/neo*^ mice at 15 and 30 days of age (Figure 6).

**Figure 7:**
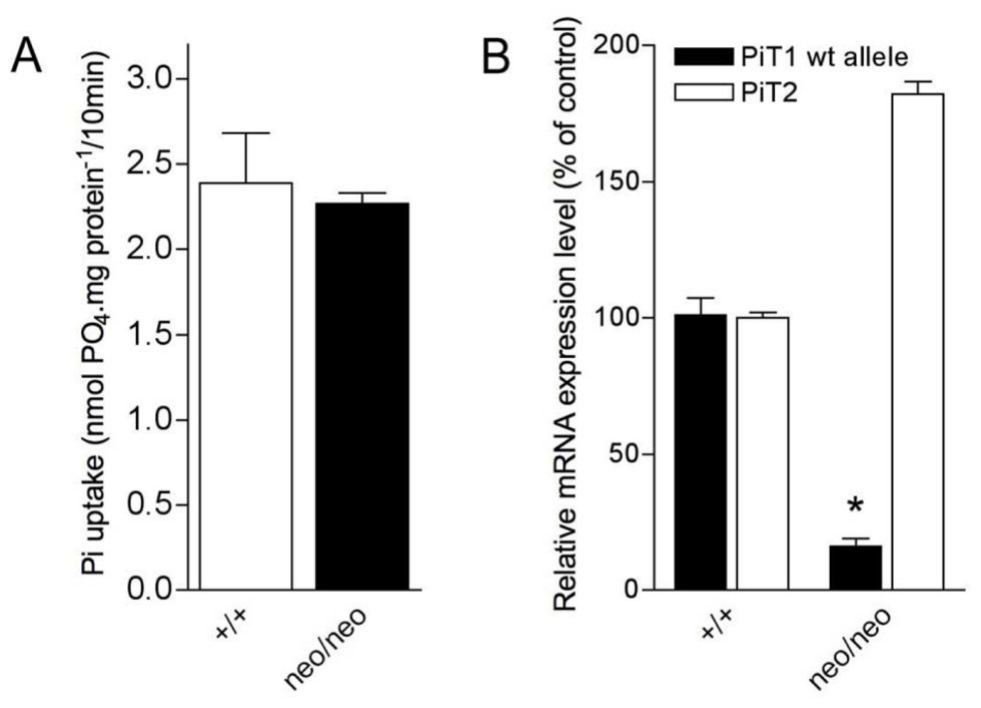
Hypomorphic expression of *PiT1* in MEFs does not affect sodium-Pi cotransport. (**A**) Sodium-Pi uptake in MEFs. The transport of Pi was measured during the linear phase of uptake using radiolabelled Pi as previously descrived [28]. Hypomorphic expression of *PiT1* in MEFs does not modify the overall Pi uptake. (**B**) Quantification of the expression of *PiT1* and *PiT2* mRNAs in *PiT1* ^*+/+*^ and *PiT1* ^*neo/neo*^ MEFs by RT-PCR. Note the 1.8-fold overexpression of *PiT2* mRNA in *PiT1*-null MEFs. * indicates significant differences as compared to wild-type controls with p=0.05, respectively.

To assess whether the *PiT2* overexpression observed in mutant animals could compensate for the decrease of *PiT1* expression, we evaluated whether this effect could affect phosphate transport in mutant cells. To assess this, we cultured mouse embryonic fibroblasts (MEFs) derived from *PiT1* ^*+/+*^ and *PiT1* ^neo/neo^ embryos, measured the relative *PiT1* and *PiT2* expression in these cells and characterized the sodium-dependent Pi uptake activity. Interestingly, our results showed that sodium-Pi uptake in *PiT1* ^*neo/neo*^ MEFs was unaffected (Figure 7A), with no change in its kinetic properties (V_max_ = 19.9 ± 0.9 and 18.7 ± 1.7 nmol.mg prot^-1^ for *PiT1* ^*+/+*^ and *PiT1* ^*neo/neo*^ MEFs, respectively; K_m_ = 120.2 ± 31.2 and 144.5 ± 6.4 mM for *PiT1* ^*+/+*^ and *PiT1* ^*neo/neo*^ MEFs, respectively). While RT-PCR confirmed that *PiT1* expression in *PiT1* ^*neo/neo*^ MEFs was decreased to 16% of wild-type value, this was associated with a 1.8-fold overexpression of *PiT2* mRNA (Figure 7B), which may account for the maintenance of normal sodium-Pi transport in *PiT1* ^*neo/neo*^ MEFs. Maintenance of a normal Pi uptake in *PiT1* ^*neo/neo*^ MEFs strengthens the hypothesis that the absence of defect in bone mineralization is likely related to the normal phosphate transport observed in bone, despite the down-regulation of *PiT1*. The normal Pi transport in MEFs, the absence of bone mineralization defect and the normal expression of *Npt* transporters are consistent with the observed normal phosphatemia of *PiT1* ^*neo/neo*^ hypomorphic mice (data not shown).

## Discussion

Many *in vitro* studies have described PiT1 as a regulated phosphate transporter in bone cells and suggested its implication in mineralization processes [15,16,18,19]. However, to date the physiological role of PiT1 in bone is unclear. In this work, we took advantage of a mouse model bearing two copies of *PiT1* hypomorphic alleles to explore the physiological relevance of PiT1 in bone mineralization. While *PiT1* ^*neo/neo*^ mice exhibit growth retardation, using bone histomorphometry, EDX and histological approaches, we could not detect significant mineralization differences between femurs of *PiT1* ^*+/+*^ and *PiT1* ^*neo/neo*^ mice of 15 to 300 days old. In particular, the growth plate of hypomorphic *PiT1* ^*neo/neo*^ femurs follows a similar age-related evolution as to wild-type femurs and exhibits a normal width and chondrocyte organization.

These results seem at odd with the impaired mineralization of 1-day-old *PiT1* ^*neo/neo*^ mice revealed by alcian blue / alizarin red S skeleton double staining and Von Kossa femur sections staining. However, it must be stressed that *PiT1* hypomorphic mice display a mild anemia at birth, which is not compensated over time [25]. As for many other mouse models, the anemia is likely to result in a temporal shift in the developmental process accounting for a growth delay of the organs. Consequently, at present we cannot discriminate between the two hypotheses. The observed mineralization differences observed at birth could either result from a decrease expression of PiT1 or a slight developmental shift (or both). Interestingly, we showed that the femurs of older hypomorphic *PiT1* mice are shorter but correctly mineralized, in accordance with a slight development delay rather than a mineralization defect. At first sight, these results are not in favor of a major role of PiT1 in bone mineralization *in vivo* and are consistent with the recent work of Suzuki and colleagues that demonstrated that although *PiT1* overexpression in transgenic rats affects the phosphocalcic metabolism, this does not modify bone mineralization nor skeletal development [30]. However, caution should be taken in making definitive statements as to the role of PiT1 in bone mineralization, especially because the mouse model used in our study does not lead to a complete PiT1 invalidation.

The 85% reduction of *PiT1* expression in hypomorphic mice was associated with a 2-fold increase in *PiT2* expression, while the expression of other Pi transporters, such as *Npt1*, *Np2a*, *Npt2b* and *Npt2c* were unchanged and comparatively very weak. Using MEFs derived from *PiT1* ^*neo/neo*^ embryos, we could show that the 2-fold increase in *PiT2* expression was enough to maintain a normal Pi uptake. Interestingly, the *PiT2* overexpression was no longer visible as the mice aged, consistent with the decrease in Pi need following the growth period of the animals [31,32]. From these results, it is tempting to speculate that the Pi uptake function bore by PiT1 can be substituted by the Pi uptake function of PiT2, leading to an absence of bone mineralization defect in *PiT1* hypomorphic mice. The exchangeable nature of the Pi transport function is plausible since PiT1 and PiT2 share almost identical uptake properties [33,34]. Hence, Pi transport rather than *PiT1* expression, may be a key function for normal bone mineralization. However, this is at variance with the observed anemia in hypomorphic mice, or the liver development defect in PiT1-null animals, which are not compensated by a *PiT2* overexpression [25].

Consistently, an alternative explanation to a PiT2-driven compensatory mechanism is the possibility that normal bone mineralization requires only small amounts of PiT1 to proceed normally, and that normal bone mineralization in hypomorphic mice may be the consequence of an incomplete *PiT1* inhibition. In favor of this hypothesis is the gene-dose effect observed in the allelic series of *PiT1* mutations in mice expressing from 0% to 100% of PiT1 [25]. Indeed, a complete knockout of *PiT1* (0% expression) leads to a lethal phenotype at E12.5 [25] that is not rescued by a *PiT2* overexpression or by any other Pi transporter. When *PiT1* expression represents 6% of wild-type value, as seen in heterozygous compound *PiT1* ^*neo/-*^ mice, embryos are able to live until E15.5 [25]. A 15% *PiT1* expression level, as in hypomorphic *PiT1* ^*neo/neo*^ mice, is sufficient to bypass the embryonic lethality but still results in a significant perinatal lethality [25]. Heterozygous *PiT1* ^*+/-*^ or *PiT1* ^*neo/+*^ mice expressing 50% of *PiT1* have a normal phenotype. This gene dose effect supports the idea that the physiological importance of PiT1 may only be revealed when its expression becomes very low. Therefore, it remains possible that the absence of bone phenotype in *PiT1* ^*neo/neo*^ mice is not due to a normalization of Pi transport following an overexpression of *PiT2*, but rather to an expression of *PiT1* that is still too high to produce bone phenotypic differences.

Nevertheless, both hypotheses are challenging the fact that PiT1 could be an essential Pi transporter for bone mineralization. Indeed, the first hypothesis relies on the existence of a functional back-up system by which the Pi-uptake function of PiT1 can be exchanged by the Pi-uptake function of PiT2, making PiT1 a dispensable Pi transporter for bone mineralization. The second hypothesis, whereby low levels of PiT1 are enough to maintain a normal bone mineralization, is not consistent with its low-capacity of Pi transport [33], making PiT1 a poor Pi transporter candidate to face the tremendous Pi needs for bone mineralization. Rather, it is possible that PiT1 possesses supplementary functions in addition to transport Pi through the membrane. In line with this hypothesis, we and others have recently identified new functions for PiT1, which are independent of its Pi-transport activity and are critical for cell proliferation and apoptosis [25,35–37]. We showed that *PiT1*-depleted MEF and HeLa cells are more sensitive to the proapoptotic activity of TNF-alpha, whereas depletion of *PiT2* had no effect [37]. The increased sensitivity to TNF-alpha apoptotic activity of *PiT1*-depleted cells was evident regardless of the presence or absence of extracellular Pi, and was blunted by re-expressing a transport-incompetent mutant of PiT1, suggesting that a defect in Pi uptake was not involved in the observed phenotype, and that the involved function of *PiT1* is unrelated to its transport activity. An apoptosis-related and Pi-transport independent function of PiT1 in bone is consistent with the discrete expression of *PiT1* in apoptotic hypertrophic chondrocytes late in development [17] whereby low levels of *PiT1* could modulate the fate of hypertrophic chondrocytes at the mineralizing front. Such a putative role of PiT1 is reinforced by the recent observation that TNF-alpha is produced by hypertrophic chondrocytes within the growth plate and that suppression of its activity leads to improved longitudinal bone growth [38]. Mice expressing low levels of *PiT1* could therefore be more sensitive to the action of TNF-alpha, consistent with the observed reduced bone length of the *PiT1* hypomorphic mice.

In summary, we report here for the first time that a low expression of *PiT1 in vivo* in mice does not affect bone mineralization. Although mineralization differences are observed in 1-day old mutant animals, this may be due to a slight developmental delay originating from the anemia. Nevertheless, this work does not rule out a role of PiT1 in bone mineralization. Rather, it points out at the actual function of PiT1 in bone that may not be related to its Pi transport properties. Accordingly, it remains to be determined whether the normal bone mineralization could be due to a functional compensation by PiT2 or to the incomplete deletion of *PiT1*. To address these questions, pertinent mouse models in which the transport functions of PiT1 and/or PiT2 are invalidated specifically in skeletal tissues are currently under investigation in our laboratory.

## Supporting Information

Figure S1 **Von Kossa and Goldner’s trichrome histological staining of femurs from *PiT1*^*+/+*^ and hypomorphic *PiT1*^*neo/neo*^ mice from 1-day to 300-days of age.**
Femurs from 1- to 300-day-old (P1 to P300) *PiT1* ^*+/+*^ and *PiT1* ^*neo/neo*^ mice were fixed in paraformaldehyde and stained using Von Kossa (**A**) or Goldner’s Masson trichrome (**B**) staining method. Using Von Kossa staining, the mineral deposition is stained in black. The Goldner’s trichrome reveals the glycoaminoglycan matrix in blue-green. With these two staining, the osteoid is stained in red. Bar represents 200 µm.
(TIF)
