# Supplementary figures and images for: Mice with Hypomorphic Expression of the Sodium-Phosphate Cotransporter PiT1/Slc20a1 Have an Unexpected Normal Bone Mineralization
Source: PLoS One. 2013 Jun 13;8(6):e65979. doi: 10.1371/journal.pone.0065979 (PMC3681848; doi:10.1371/journal.pone.0065979)

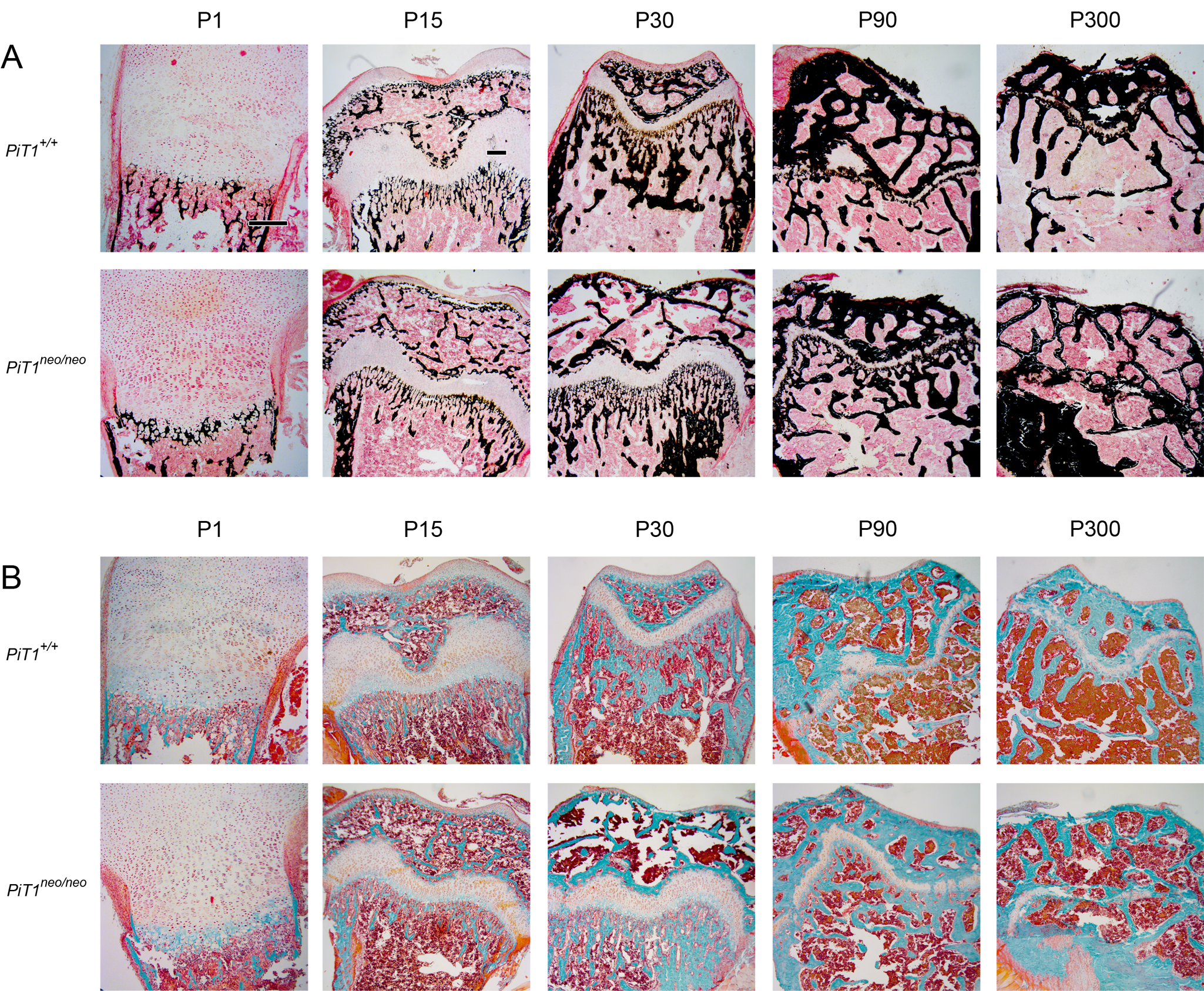

Supplement: Figure S1 — Von Kossa and Goldner’s trichrome histological staining of femurs from PiT1+/+ and hypomorphic PiT1neo/neo mice from 1-day to 300-days of age. Femurs from 1- to 300-day-old (P1 to P300) PiT1 +/+ and PiT1 neo/neo mice were fixed in paraformaldehyde and stained using Von Kossa (A) or Goldner’s Masson trichrome (B) staining method. Using Von Kossa staining, the mineral deposition is stained in black. The Goldner’s trichrome reveals the glycoaminoglycan matrix in blue-green. With these two staining, the osteoid is stained in red. Bar represents 200 µm. (TIF) [file pone.0065979.s001.tif]
